# A Simplified Method for the Preparation of Highly Conductive and Flexible Silk Nanofibrils/MXene Membrane

**DOI:** 10.3390/ma16216960

**Published:** 2023-10-30

**Authors:** Bohan Ding, Chao Teng, Yanxiang Wang, Yongbo Wang, Haotian Jiang, Yue Sun, Jinghe Guo, Shichao Dai

**Affiliations:** 1 Carbon Fiber Engineering Research Center, School of Materials Science and Engineering, Shandong University, Jinan 250061, China; 2 College of Materials Science and Engineering, Qingdao University of Science and Technology, Qingdao 266042, China

**Keywords:** silk nanofibrils, solution stripping, nanofibrils, nanocomposite material

## Abstract

Silk nanofibers (SNF) have great applications in high-performance functional nanocomposites due to their excellent mechanical properties, biocompatibility, and degradability. However, the preparation of SNF by traditional methods often requires the use of some environmentally harmful or toxic reagents, limiting its application in green chemistry. In this paper, we successfully prepared SNF using natural silk as raw material and solvent stripping technology by adjusting the solvent concentration and solution ratio (the diameter of about 120 nm). Using the above SNFs as raw materials, SNF membranes were prepared by vacuum filtration technology. In addition, we prepared an SNF/MXene nanocomposite material with excellent humidity sensitivity by simply coating MXene nanosheets with silk fibers. The conductivity of the material can approach 1400.6 S m^−1^ with excellent mechanical strength (51.34 MPa). The SNF/MXene nanocomposite material with high mechanical properties, high conductivity, and green degradability can be potentially applied in the field of electromagnetic interference (EMI) shielding, providing a feasible approach for the development of functional nanocomposite materials.

## 1. Introduction

In recent years, nanocomposites have attracted a lot of attention from researchers due to their excellent functionality and ease of processing. Influenced by the concept of sustainable development, more and more researchers are committed to finding new environmentally friendly materials with high performance [1]. Flexible membranes obtained from nanomaterials by vacuum filtration and casting are widely used to prepare functional materials [2,3]. Herein, we set our eyes on nature because, with thousands of years of evolution and natural selection, natural biomaterials have evolved a quantity of impressed functional materials with specific construction, such as spider silk [4], lotus leaves [5], and natural silkworm silk. Silk nanofibril (SNF), as an organic matter, with favorable degradability [6,7] coincides with the concepts of green chemistry and sustainable development, and, at the same time, its high mechanical properties [8,9,10,11,12] and outstanding biocompatibility [13] make it have great potential in the fields of tissue engineering [14,15,16], drug delivery [17], electronic materials [7,18,19], membrane separation technology [20], biomedical devices [21] and hematology [22,23].

Silk is composed of two main proteins: silk fibroin (SF) and sericin. SF provides mechanical strength and is semi-crystalline, while sericin is mainly amorphous and acts as a glue to encapsulate SF to maintain the structural integrity of the cocoon [24]. The high mechanical strength of silk can be attributed to its chemical composition, structural hierarchy, and hydrophobic domains that facilitate antiparallel β-sheet crystal formation [25].

Natural silk can be degummed in a variety of ways to obtain SF. Degumming can usually take place with the use of urea [26], neutral soaps [27], proteases [28,29,30], high-temperature and pressure treatments [31], autoclaving [32] and boiling in alkaline solutions [33]. Liu et al. investigated the effect of several natural proteases on silk degumming and showed that the degumming rate of silk using natural proteases was much lower than the silk using Na_2_CO_3_ [30]. Gaviria et al. explored the differences in morphology, mechanical properties, amino acid content, etc., of SF degummed by the autoclaving method and the chemical method [32]. In addition, the use of different reagents had an effect on the rate of membrane degradation, with the degradation rate of membranes degraded by the acidic solvent being lower than that using the alkaline solvent [34].

To date, a great deal of research on silk has focused on regenerated silk fibroin (RSF), which can be produced via a bottom-up process, such as dissolving silks with LiBr or HFIP and then regenerating it by electrostatic spinning [35], dry spinning [36] and wet spinning [37], etc. Specifically, in the case of LiBr, the SF was first completely dissolved in LiBr solution. The obtained solution was dialyzed in a dialysis bag to remove salt ions. Further centrifugation is performed to remove insoluble substances, at which point the solution obtained is a pure RSF solution. The solution can be further subjected to use, such as electrostatic spinning and freeze-drying to achieve a variety of nanoscale products [38]. These methods make it easy to produce nanoscale fibers with excellent yield. However, this dissolution–regeneration process greatly destroys the hierarchical structure of SF and inevitably sacrifices the natural properties of SF [39]. SNFs, as the basic unit of silk fine structure, possess all the excellent properties of silk and become a potential candidate for functional nanocomposites.

In contrast, SNF can be produced from natural silk in a top-down process such as treatment with HFIP [40] or NaClO [41] followed by ultrasonication, but these process needs to use of toxic solutions. Therefore, a green preparation method was used to produce SNFs using a mixed solution of CaCl_2_, C_2_H_5_OH and H_2_O to treat the SF, which was then subjected to high-speed shearing [39].

As a recent emerged two-dimensional (2D) transition-metal carbides and/or nitrides [42], MXene has been widely used in functional materials based on its unique structure [43] and excellent electrical properties [44]. Since Y. Gogotsi et al. first reported the application of MXene in EMI shielding in 2016 [45], MXene has been widely noted for its low density and ease of processing compared to metal materials [46,47]. Currently, the main preparation method of Mxene is to be prepared by selectively etching the A atomic layer from the precursor MAX powder in a mixed LiF/HCl solution according to the minimum intensity layering (MILD) method [48].

Notably, the surface terminations of MXene are strongly influenced by the method of MXene‘s preparation. And the differences in surface terminations in turn directly affect the physical and chemical properties of MXene [49]. When we prepared MXene by etching MAX powder with LiF/HCL solution, a significant number of functional groups such as -F, -O and -OH exist on the surface of MXene [46,50]. This facilitates the formation of hydrogen bonds between MXene and other polymer matrices, including PVA [51], cellulose nanofiber (CNF) [52] and sodium alginate (SA) [53], and also can easily bind to -NH and -OH on the surface of proteins for use as bio-nanomaterials [54].

Up to now, the main applications of SNF are still concentrated in the biomedical field, and there is still a lack of research on the preparation of high-performance composites by combining SNF with conductive 2D materials such as MXene and graphene. Meanwhile, most of the current studies on silk have focused on the application of RSF [25], while there are still fewer reports on SNF. Given the excellent flexibility of SNF and its outstanding application in sensors, the study attempts to composite SNF with MXene to obtain nanocomposites with high conductive and mechanical properties to have a wide range of applications in the fields of EMI shielding and electro-thermal conversion.

## 2. Materials and Methods

### 2.1. Material

Bombyx mori cocoons were purchased from Ankang, (Shaanxi, China). Calcium chloride anhydrous (CaCl_2_) and sodium carbonate (Na_2_CO_3_) were purchased from Sinopharm Co., Ltd. (Shanghai, China). Ethanol (C_2_H_5_OH, 99.5%) was obtained from Macklin Biochemical Co., Ltd. (Shanghai, China). MXene dispersion was prepared in the laboratory.

### 2.2. Preparation of SNF/MXene Composite Membrane

Bombyx mori cocoons were taken and cut into slices, and the slices of Bombyx mori were immersed into the Na_2_CO_3_ solution (bath ratio of 1:50, g mL^−1^), and heated in a water bath at 100 °C. This process was repeated three times in order to obtain the degummed SF. The dried SF fiber was treated with mixed solution (the molar ratio of CaCl_2_:CH_3_CH_2_OH: H_2_O was 1:2:8; bath ratio of 1:100, g mL^−1^) at 45 °C for 6 h. We removed the solid fraction, washed it at least 6 times with deionized water to ensure complete removal of salt ions and then dried it in an oven at 70 °C. Then, SF was put in high-speed shear (HAY-8105, Guangdong, China), added appropriate amount of deionized water and sheared for 15 min, the clear liquid part is SNF suspension. The SNF suspension was filtered using filter paper with a 0.2 μm aperture to obtain the SNF membrane. Different masses of MXene solution were taken and uniformly coated on the surface of SNF membrane to prepare 40 wt%, 60 wt% and 80 wt% SNF/MXene membranes, which were put into a vacuum drying oven at 40 °C and dried for 24 h, i.e., SNF/MXene membranes with different mass ratios were obtained.

### 2.3. Characterization

The morphology and structure of the samples were investigated by scanning electron microscopy (SEM, Regulus 8100, Hitachi, Tokyo, Japan). Fourier transform infrared spectroscopy (FTIR, VERTEX 70, Bruker, Berlin, Germany) was used to judge whether the SNF and MXene were successfully composited or not. For the performance of the composite membrane, the universal tensile testing machine (KXWW-05C, K-TEST, Chengde China) was used to carry out mechanical testing, to obtain the stress–strain curve of the composite membrane with different mass ratios. The square resistances of SNF/MXene composite membranes with varying ratios of mass were measured using a multifunctional digital four-probe tester (ST-2258C, JG, Suzhou, China), and their conductivity was calculated according to the following equation:

(1) σ=R/d

where *σ* is the membrane conductivity, R is the measured square resistance, and d is the membrane thickness.

## 3. Results

### 3.1. Fabrication and Characterization of SNFs

**Figure 1:**
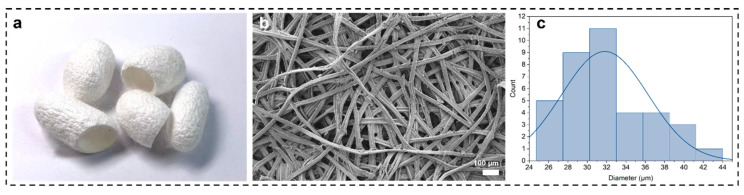
(**a**) Digital image of Bombyx mori cocoons, (**b**) SEM image of silk protofibrils, (**c**) statistical graph of the diameter of the protofibrils of silkworms.

Figure 1 shows the morphology of the original silk. The digital image of the cocoons, shown in Figure 1a, are spindle shaped, with a bright white surface accompanied by irregular wrinkles and a tough, lustrous texture, made of layers of silk attached to the cocoons. SEM characterization was performed as in Figure 1b, which shows that a bundle of silk fibrils is composed of two piles of SF wrapped by sericin, and we counted the diameters of the silk protofibrils as in Figure 1c, which ranged from 20 to 40 μm in diameter and the mean diameter of the fibers is 31.83 μm.

**Figure 2:**
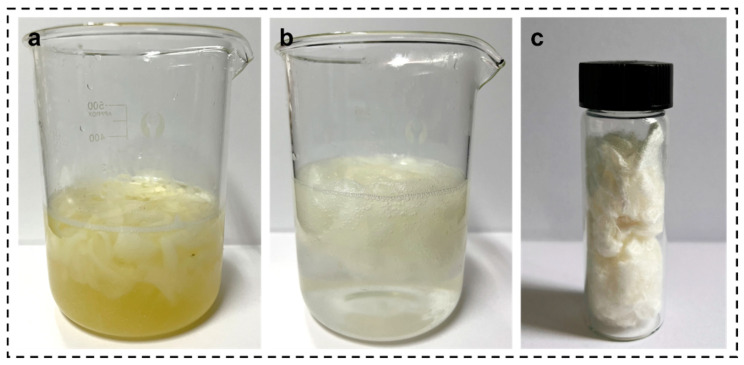
(**a**) Digital image of the first degumming system, (**b**) digital image of the third degumming system, (**c**) digital image of the degummed SF.

**Figure 3:**
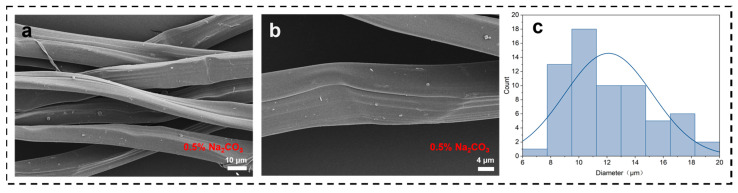

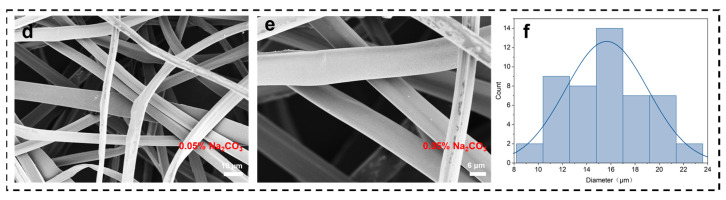
(**a**) SEM image of SF-0.5 (**b**), SEM image of a single SF-0.5, (**c**) distribution charts of SF-0.5 diameter, (**d**) SEM image of degummed SF-0.05, (**e**) SEM image of degummed SF-0.05, (**f**) distribution charts of SF-0.05 diameter.

Figure 2 and Figure 3 illustrate the degumming of SF. Figure 2 shows the relationship between the number of degumming times and the color of the solution: when the Na_2_CO_3_ solution was used for the first boiling, the solution was yellow as shown in Figure 2a, and when three times degumming was carried out, the color of the solution was colorless and transparent as shown in Figure 2b.

To determine the optimum alkali solution concentration for silk degumming, SF was degummed by using 0.5 wt% (SF-0.5) and 0.05 wt% (SF-0.05) Na_2_CO_3_ for control as shown in Figure 3. By comparing the above results, Figure 3a,b show that SF-0.5 has a great deal of damage, while SF-0.05 is smooth and regular. Thus, 0.05 wt% Na_2_CO_3_ solution was selected for further investigation. At the same time, the diameters of degummed SF were mainly distributed around 12 μm, the mean diameter of the degummed SF is 12.11 μm., which was basically half of the diameter of the silk fibrils, and could also prove the hypothesis of the structure of the silk fibrils we made earlier.

Traditional methods of obtaining SNFs require the use of some toxic solvents, such as HFIP and NaClO with ultrasonication, and the preparation of RSF also requires the LiBr to dissolution; these solvents are all toxic and also have pollution problems to the environment. Therefore, a green method was used for the preparation of SNFs in this paper. SF was treated using a mixed solution to attenuate the force between the fibrils. This process can create small gaps between the fibrils. Meanwhile, the addition of C_2_H_5_OH molecules enhanced the movement of the hydrophobic side chains of the silk protein. More importantly, the Ca^2+^ ions can combine with the carbonyl oxygen on the filipin protein chains, breaking the hydrogen bonds between the filipin protein chain segments, and at the same time, weakening the hydrophobicity of the chain segments, accelerating the solution swelling rate. After pretreatment by solution, SNF can be easily obtained after mechanical shearing.

**Figure 4:**
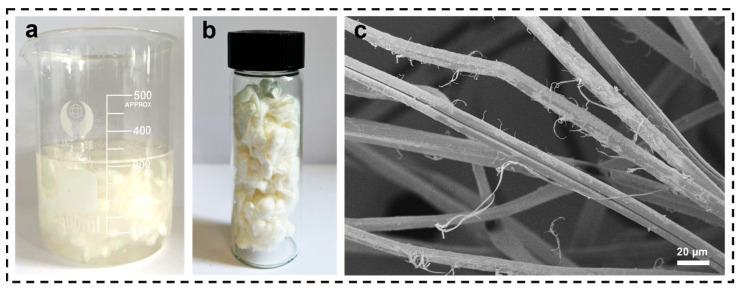
(**a**) Digital image of SF pretreated with mixed solution, (**b**) SF pretreated with mixed solution, (**c**) SEM image of pretreated SF with mixed solution.

To further investigate the morphology of the pretreated SF, SEM characterization was carried out in this paper. As shown in Figure 4c, it can be seen that a small amount of separation of microfibrils occurred in the pretreated SF compared to the degummed SF, which is related to the solution swelling and the disruption of hydrogen bonding by Ca^2+^.

**Figure 5:**
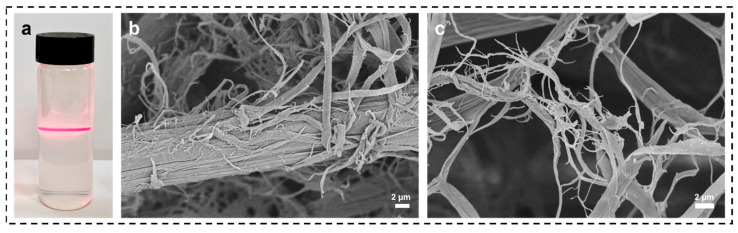
(**a**) Tyndall effect of SNF solution, (**b**) SEM image of SF mechanically stripped for 1 min, (**c**) SEM image of SF mechanically stripped for 15 min.

Dry the SF after the above swelling treatment, put it in the high-speed shear, add an appropriate amount of deionized water for mechanical stripping, and strip it for 15 min to obtain the SNF solution. As shown in Figure 5a when laser light was transmitted through the SNF solution, the Tyndall effect was clearly observed, which proved that the fibrils in the solution were dispersed to the nanoscale. Meanwhile, the SF taken from mechanical shearing for 1 min and mechanical shearing for 15 min were subjected to SEM characterization as shown in Figure 5b,c, which shows that the silk protofibrils in mechanical shearing for 1 min have shown the exfoliation of micrometer-sized filaments. In contrast, the filaments in shearing for 15 min can be observed to have obtained a certain number of nano-scaled fibrils.

**Figure 6:**
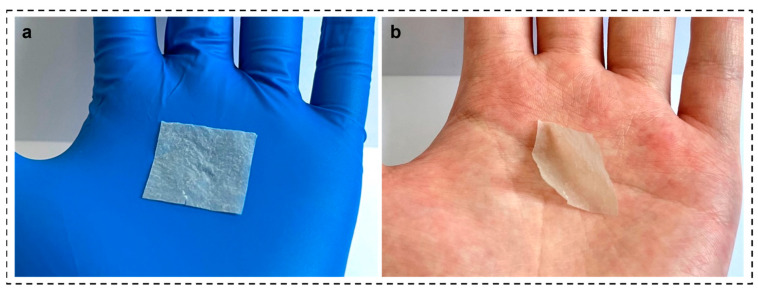

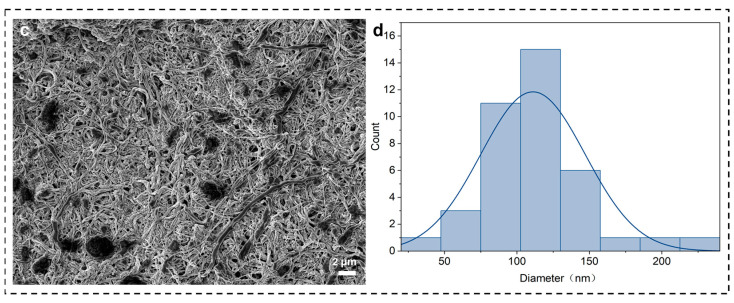
(**a**) SNF film exhibits flat spreading in gloved hands, (**b**) bending response in wet hands, (**c**) SNF film SEM image, (**d**) SNF diameter statistic charts.

The SNF solution was vacuum-evacuated through a mixed cellulose filter membrane with a diameter of 50 mm and a pore size of 0.2 μm, and the SNF membrane was obtained after drying at room temperature. As shown in Figure 6a,b, the SNF membrane was observed to have excellent moisture-sensitive response properties when the SNF membrane was placed in wet hands and the membrane rapidly curled inwards within a second, while in dry rubber gloves, the membrane adhered to the surface of the gloves. Upon placing the membrane in a wet and dry environment, it spontaneously returns to its original flat state. Since SF are composed of different hydrophilic and hydrophobic amino acids, it is speculated that this bending response is due to the water molecules in the humid environment forming new hydrogen-bonding interactions with polar groups (-NH, -OH, C=O) in certain amino acids, leading to this rapid moisture-sensitive response. In summary, it can be concluded that SNF membranes can be used as a more stable moisture-sensitive response material. To further observe the surface morphology of the SNF membrane, the membrane was characterized by SEM, as shown in Figure 6c, the membrane was made of a large number of fine nano-scaled fibrils interwoven and stacked. Meanwhile, the diameter distribution of the fibrils was counted as in Figure 6d; the diameter was mainly concentrated around 120 nm, and the distribution range was within 35–250 nm, with an average diameter of 111.02 nm.

### 3.2. Preparation and Characterization of SNF/MXene Composite Membrane

**Figure 7:**
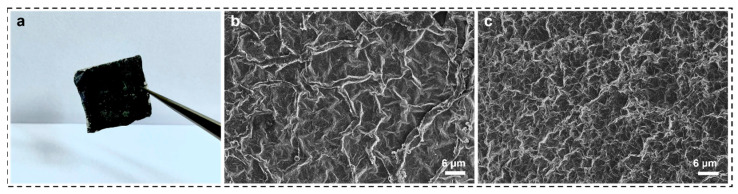
(**a**) Optical photograph of SNF/MXene film, (**b**) SEM image of 60% MXene film, (**c**) SEM image of 80% MXene film.

SNF/MXene was prepared by the drop-coating method, as shown in Figure 7a, the surface of the composite membrane was black with a certain flexibility, in order to further observe its surface structure, SEM characterization was carried out for the composite membranes with different concentrations respectively. Figure 7b,c are the SEM images of the composite membranes with MXene content of 60% and 80%, respectively, and obvious MXene composite membrane crumpled characteristics can be observed. The obvious crumpled characteristics of MXene composite membranes were observed, and the difference between the two was that the crumpled density was different, and the crumpled density of the composite membrane with 60% MXene content was obviously lower than that of the composite membrane with 80% MXene content, and the thickness of the membranes was around 10 μm by SEM characterization.

**Figure 8:**
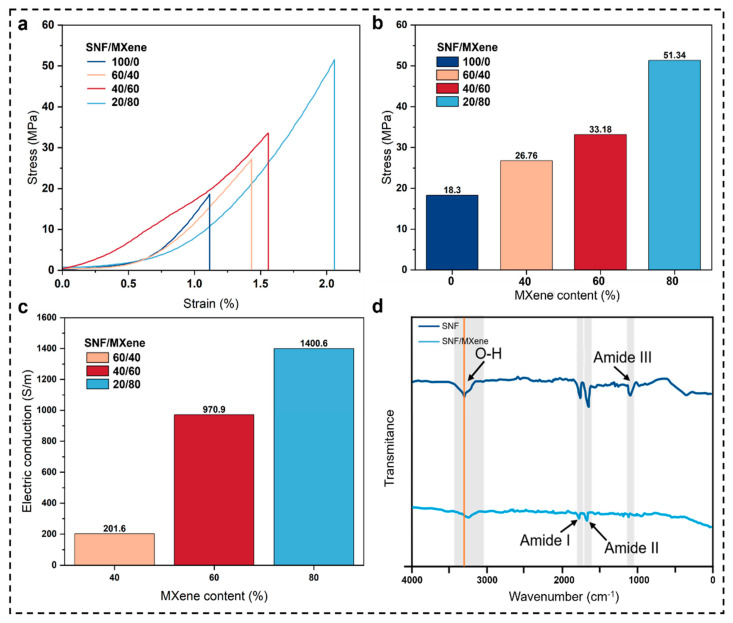
(**a**)Tensile stress-strain curves of composite films with different SNF and MXene weight ratios, (**b**) maximum stress diagram of composite films with different SNF and MXene weight ratios, (**c**) conductivity of SNF/MXene films with different MXene content, (**d**) FTIR spectra of pure SNF film and SNF/MXene composite film.

The mechanical properties of pure SNF membrane and SNF/MXene membrane were tested using a universal tensile testing machine. As shown in Figure 8a, the SNF membrane is brittle and therefore has a lower strain. Meanwhile, it can be observed that the stress of the pure membrane is the lowest, which is about 18.3 MPa, and the stress of the composite membrane increases as the content of MXene increases, and the stress of the membrane can reach 51.34 MPa when the content of MXene reaches 80%, which may be related to the formation of hydrogen-bonding interactions between the groups such as -OH and -F in MXene and -NH and C=O on the surface of SNF.

The square resistance value of the composite membrane was tested using a four-probe tester and its conductivity value was calculated, as shown in Figure 8c, the SNF/MXene composite membrane shows an increase in conductivity with the increase in MXene content. The membrane conductivity was 201.6 S m^−1^ at 40% MXene content and 970.9 S m^−1^ at 60% MXene content. The conductivity can be as high as 1400.561 S m^−1^ when the MXene content is controlled to 80%.

To confirm that MXene and SNF have been complexed, the pure SNF membrane and SNF/MXene composite membrane were characterized by FTIR. As shown in Figure 8d, the absorption peaks at wavelengths of 1618~1620 cm^−1^, 1506~1508 cm^−1^ and 1223~1225 cm^−1^ corresponded to Amide I, Amide II and Amide III in the secondary structure of nanofilament protein, respectively. Amide I and Amide II are the characteristic peaks of the β-sheet in the secondary structure of the protein, and Amide III is the peak of the β-sheet in the secondary structure of the protein. Amide III is the characteristic peak of α-helix in the protein secondary structure. It is easy to see that the intensities of the Amide I and Amide II peaks of SNF/MXene composite membranes are significantly lower than those of pure SNF membranes, which may be attributed to the formation of a large number of hydrogen bonds between MXene and the surface of the filipin protein due to the incorporation of MXene, and also makes it difficult to transform the random clusters of protein chains and α-helical structure to β-sheet structure, and this confirms that the intensity of the peaks of the α-helical structure of protein secondary structure is lower with the increase in the content of MXene in the mechanical property test. This also confirms the conclusion that the membrane stress increases with the rise of MXene content in the mechanical property test. In addition, the wavelength of 3276–3280 cm^−1^ corresponds to the characteristic absorption peak of O-H bonding, which is red-shifted to a certain extent in the SNF/MXene composite membrane compared with the pure SNF membrane. This proves the existence of hydrogen bonding between MXene and SNF.

## 4. Conclusions

In this study, SNFs were prepared by three steps of degumming, pretreatment and mechanical stripping, and successfully obtained a natural SNF solution with high dispersion and a mean diameter of 111.02 nm. The solution was filtered to obtain a transparent SNF membrane with flexibility, and the fast moisture-sensitive response characteristics of SNF were observed, so SNF can be applied as a stable moisture-sensitive sensor. The obtained membranes were laminated with MXene to obtain SNF/MXene membranes with excellent conductivity. In order to further investigate the resulting material, tested its mechanical and electrical properties and characterized it with infrared tests. Specifically, the tensile test of the composite membrane was carried out using a universal tensile testing machine, and the results showed that the stress of SNF/MXene composite membrane increases with the increase in MXene content, and the maximum stress is 51.34 MPa when the MXene content is 80%. The four-probe tester was used to measure the conductivity of the membrane, which showed that the SNF/MXene membrane has excellent electrical conductivity, and with the increase in MXene content, the conductivity also increases gradually, and its conductivity can reach up to 1400.561 S m^−1^. To sum up, these advantages of SNF/MXene composite film can make it widely used in flexible electronic devices and so on; moreover, due to the good biocompatibility, environmental friendliness and degradability of SNF, SNF will play an important role in the field of EMI shielding, sensors and so on.

## Data Availability

Not applicable.
